# Stahl’s ear correction: A review of surgical approaches

**DOI:** 10.1016/j.jpra.2025.07.001

**Published:** 2025-07-11

**Authors:** Aref Nassar, Joy Naba, Dollen Eid, Samer Abou Zeid, Joe Demian

**Affiliations:** a Plastic Surgery Department, Hotel Dieu de France, Saint Joseph University, Beirut, Lebanon; b Dermatology Department, Hotel Dieu de France, Saint Joseph University, Beirut, Lebanon; c Saint Joseph University, Beirut, Lebanon

**Keywords:** Stahl’s ear, Stahl ear, Third crus, Congenital ear deformity, Auricular malformation, Auricular cartilage, Ear surgery

## Abstract

**Background:**

Stahl’s ear is a congenital deformity involving an additional third crus and a pointed upper helix, leading to aesthetic and functional concerns. Surgical correction is often necessary for severe cases or when nonsurgical methods are ineffective. However, there is limited comprehensive guidance on surgical technique selection.

**Objectives:**

This review evaluates the full spectrum of surgical techniques for Stahl’s ear correction, aiming to provide a comparative framework for surgical decision-making.

**Methods:**

A systematic search of PubMed, MEDLINE, and Embase databases identified 18 studies published through October 2024. Data on surgical techniques, outcomes, and complications were extracted and synthesized.

**Results:**

The review analyzes surgical techniques for Stahl’s ear correction, evaluating their advantages, limitations, and clinical applications. Surgical methods were categorized into cartilage-sparing and cartilage-cutting techniques, with combined approaches also discussed. A comprehensive comparison of techniques is provided, consolidating scattered data into a practical guide for tailoring treatment to patient-specific anatomy.

**Conclusions:**

Surgical correction of Stahl’s ear requires a patient-centered approach, balancing deformity severity and cartilage characteristics. This study presents the first comprehensive review of surgical techniques for correcting Stahl’s ear deformity, providing a detailed analysis of their advantages, limitations, and clinical indications. Further research is needed to evaluate long-term outcomes and refine evidence-based guidelines.

## Introduction

**Figure 1:**
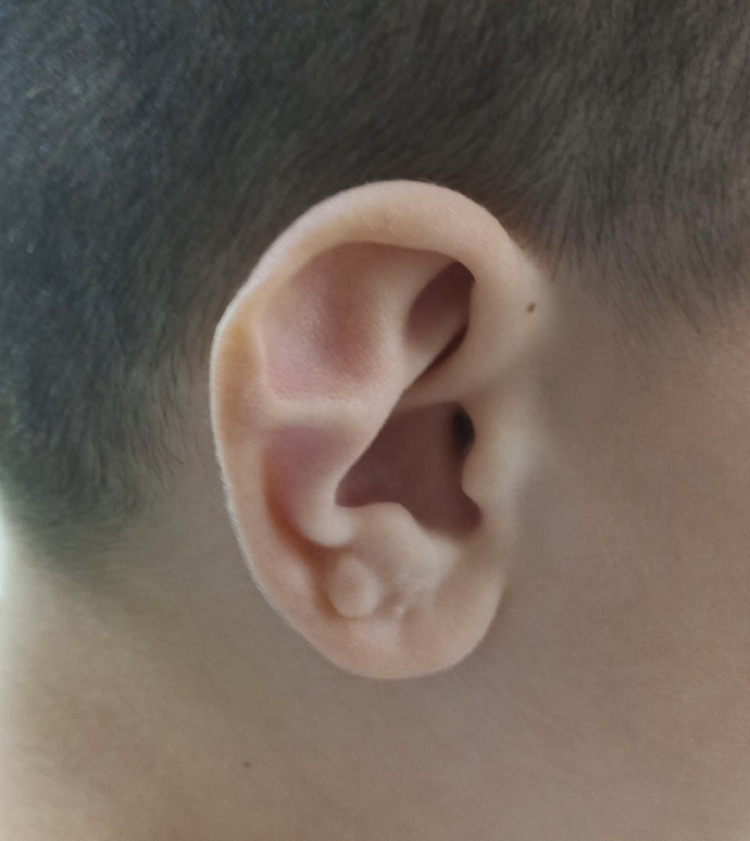
Photograph of a patient with Stahl’s ear deformity.

Stahl’s ear is a congenital ear malformation characterized by an additional, horizontally oriented third crus and a pointed upper helix (Figure 1). Other deformities frequently accompany Stahl's ear, including a missing superior crus, a narrowed helix, and widening of the scaphoid fossa.^1^

Although typically unilateral, it presents bilaterally in approximately 20% of cases.^2^ This deformity is thought to result from an abnormal insertion of the transverse auricular muscle.^3^ To better understand its variations, Yamada et al. classified Stahl’s ear deformities into four groups based on the characteristics of the third crus.^4^ In Group 1, the third crus extends posterosuperiorly, forming a sharp ridge; in Group 2, it extends similarly but with a round ridge. Group 3 features a posterosuperior extension creating two ridges, while in Group 4, the third crus extends posteroinferiorly from the antihelices.

Nonsurgical correction techniques have shown efficacy in treating Stahl’s ear with low complication rates.^5^^,^^6^ However, delayed presentation may preclude nonsurgical options, and in some cases, these methods cannot adequately address cartilage deformity. For such cases, surgical correction becomes necessary. Various surgical techniques have been described to address Stahl’s ear malformation. To date, no comprehensive review has analyzed the full range of surgical techniques for Stahl's ear correction. This review is the first to present existing methods, examining their advantages and drawbacks to determine which technique may be best suited to each patient’s specific presentation.

## Methods

A comprehensive literature search of PubMed, Medline, and Embase databases was conducted for studies through October 2024, focusing on techniques and outcomes for the surgical correction of Stahl's ear deformity. The search included the terms “Stahl's ear,” “congenital ear deformity,” “third crus correction,” “ear malformation surgery,” and “auricular cartilage reconstruction.”

Two reviewers independently screened the titles, abstracts, and full texts of the identified articles. Additional relevant studies were identified by reviewing the reference lists of initially selected articles. Data were extracted on surgical techniques, patient outcomes, and complications. Articles lacking detailed information on Stahl's ear or its surgical correction methods were excluded from the analysis.

## Results

A total of 30 potentially relevant publications were identified through the database search. After a detailed review of the full texts, 18 studies were deemed relevant, as they provided comprehensive descriptions of surgical techniques for correcting Stahl's ear deformity. Table 1 provides a summary of the various studies and surgical techniques.

**Table 1:** Summary of studies on surgical correction of Stahl's ear.

Year	Author	Article	Technique	Number of patients
1980	Yamada	Evaluation of Stahl's ear, Third crus of antihelix	Excision of third crus + horizontal mattress sutures	38
1984	Nakajima	Surgical and Conservative Repair of Stahl's Ear	3 techniques to remove 3rd crus + mattress sutures for upper crus creation	18
1985	Furukawa	A simple operative procedure for the treatment of Stahl’s ear	Posterior incision along third crus + inverting sutures	1
1985	Nakayama	Surgical treatment of Stahl's ear using the periosteal string	Horizontal mattress sutures + periosteal string to correct helical rim deformity	4
1989	Sugino	Surgical correction of Stahl's ear using the cartilage turnover and rotation method	Circular cartilage removal and rotation	3
1992	Tsujiguchi	A new method for correction of Stahl's ear	Rectangular cartilage flap incorporating the third crus advanced toward helical rim	5
1994	Noguchi	Simple surgical correction of Stahl’s ear	Deformed auricular cartilage removed then cut into pieces and grafted	5
1996	Ono	An operation for Stahl’s ear	Wedge excision of third crus and abnormal helix + posterior cartilage graft and cartilage suture	5
1998	Kaplan	A novel surgical method of repair for Stahl's ear	Third crus excision + onlay graft in the anatomical position	1
2004	Al-Qattan	An Alternative Approach for Correction of Stahl’s Ear	Third crus excision + two cartilage grafts	5
2008	El Kollali	Posterior Z-plasty and J-Y antihelixplasty for correction of Stahl’s ear deformity	Posterior skin Z plasty and mattress sutures	5
2010	Liu	A new method to correct Stahl’s ear	Cartilage scoring + folded flap	17
2012	Weinfeld	Stahl’s Ear Correction: Synergistic Use of Cartilage Abrading, Strategic Mustarde Suture Placement, and Anterior Anticonvexity Suture	Abrading + suture method	2
2013	Gundeslioglu	Stahl Ear Correction Using the Third Crus Cartilage Flap	Cartilage flap to create superior crus + excision of extra skin and cartilage	4
2016	Min Kim	Innovative method to correct Stahl ear that involves full thickness scoring incisions and an onlay graft of cymba conchal cartilage	Posterior cartilage scoring + cartilage graft	1
2017	Borrelli	A Novel Three-Step Method for Correction of Type 1 Stahl Ear	Anterior excision of wedge + horizontal mattress suture for superior crus creation	1
2019	Sinnott	The Double-reverse Wedge Excision Technique: A Novel Approach to Reconstruction of Stahl’s Ear Deformity	Wedge excision of third crus + mattress sutures for superior crus + opposing skin wedge excision	1
2020	Kazi	Surgical correction of Stahl ear using cartilage-cutting and -sparing techniques	Cartilage scoring and suture placement	1

Petersson categorized surgical methods for correcting Stahl’s ear into two main types: cartilage-sparing and cartilage-cutting techniques.^7^ In this review, we present a detailed examination of these techniques, beginning with cartilage-sparing approaches.

### Cartilage-sparing techniques

These techniques rely on sutures and cartilage repositioning to reshape the ear, without any cartilage excision. The techniques involving cartilage scoring and suture placement include:

Furukawa^8^ described a technique where a posterior incision parallel to the helix is made, followed by subperichondrial dissection. Posterior incisions are performed along the third crus, and a horizontal mattress suture is placed to invert the helical cartilage. This maneuver corrects the protruding helix and creates a concave shape in the scapha without forming a superior crus.

Weinfeld’s^9^ correction of the anomaly involves three steps. First, through a retroauricular incision and dissection, posterior cartilage abrasion is performed, focusing on the area corresponding to the third crus. Additionally, anterior abrasion of the superior crus is achieved through a cartilage fenestration. This abrasion helps relieve interlocking stresses within the cartilage, promoting controlled directional bending.

Next, Mustardé sutures are placed from the posterior scapha, at the point of maximal convexity of the third crus, to the posterior concha. These sutures aid in flattening the third crus’s convexity while enhancing the superior crus’s shape by increasing its angulation and anterior convexity when tightened.

Finally, through an anterior approach with an incision over the third crus, horizontal mattress sutures are placed cephalically and caudally to the third crus, further reducing its convexity.

The authors add that if preoperative assessment shows that the scapha has a square perimeter shape with a wide triangular fossa, an excisional approach may be considered to address these features, as this technique alone may not be sufficient for their correction.

**Figure 2:**
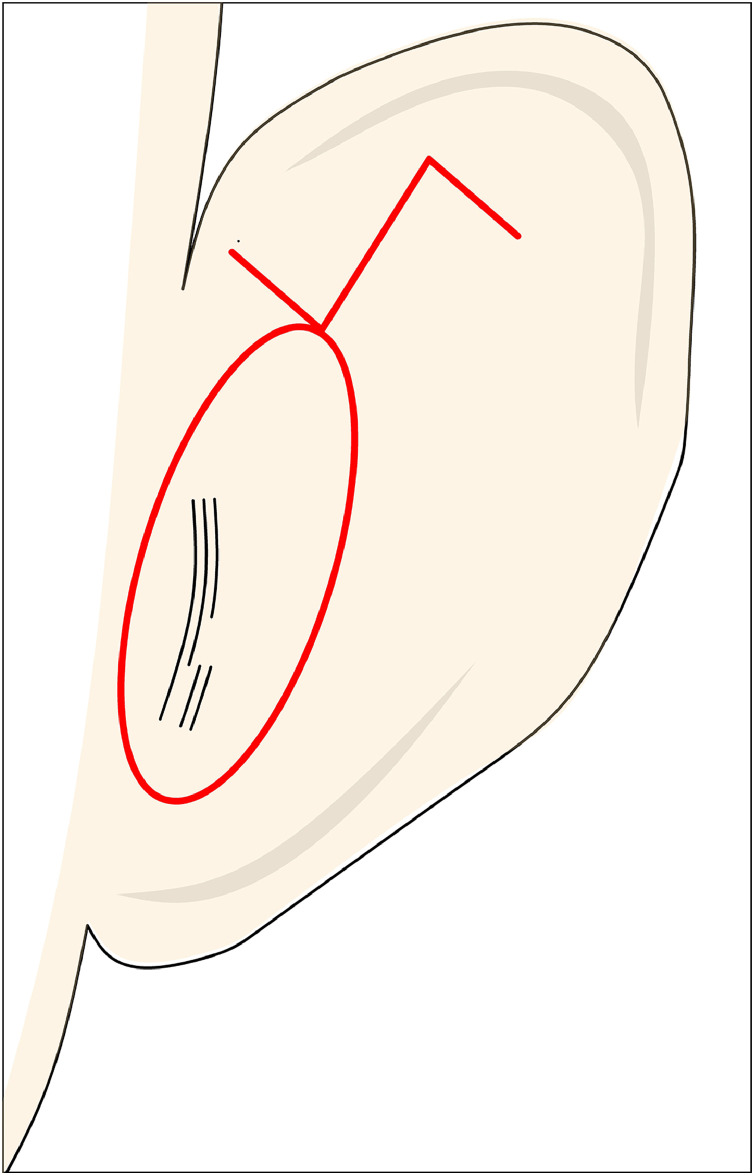
Figure depicting El Kollali’s technique, showing posterior markings on the ear for the elliptical incision and the Z-plasty. Note that the long arm of the Z is positioned perpendicular to the planned location of the superior crus.

El Kollali^10^ proposed a technique where the position of the superior crus is marked using a needle impregnated with methylene blue, which is passed from anterior to posterior to mark these points on the cartilage. A postauricular wedge incision is then made, with the superior part designed as a Z-plasty, where the long axis of the Z is perpendicular with the third crus (Figure 2).

During dissection, an abnormal auricular muscle may be encountered, which should be incised to aid in correcting the deformity. The posterior surface of the third crus is scored, and figure-of-8 sutures are placed from the posterior surface of the third crus to a point medial to the antihelix. These sutures help to flatten the third crus and create a natural-looking upper crus.

The Z-plasty of the postauricular skin addresses the skin shortage in the area of the third crus, effectively increasing vertical height while reducing horizontal width, thus contributing to the correction of the deformity in Stahl’s ear. However, helical rim irregularities are not corrected in this technique.

Kazi^11^ described another technique: In the operating room, manual flattening of the third crus is performed to create a superior crus, which is then marked and scored percutaneously with a needle. A postauricular incision is made, followed by posterior skin dissection. The third crus is scored, and Mustardé sutures are placed at the location of the previously marked superior crus to form it. This process creates a normal-appearing crus while flattening the original third crus of Stahl’s ear.

If the third crus remains prominent, additional dissection is performed anteriorly. The third crus cartilage is then scored, and reverse Mustardé sutures are applied to the anterior surface to further flatten it.

This cartilage-sparing technique is suitable for infants with malleable cartilage. However, if the cartilage is thick and resistant, this method may not adequately correct the deformity.

Next, the techniques involving cartilage reshaping through cartilage incisions, grafts, or flaps without cartilage excision include:

Noguchi^12^ described a straightforward technique for correction, followed by an extended period of ear splinting. A postauricular incision and subcutaneous dissection are performed to expose the deformed cartilage, which is then excised. The removed cartilage is sectioned into smaller pieces and grafted into a perichondrial pocket, followed by closure of the perichondrium and skin. This approach creates an amorphous cartilage state that effectively corrects the auricular deformity. The helix is shaped into a normal ear contour and secured with a bolster for one week, while a splint is applied to help mold the scapha and helix, remaining in place for one month.

**Figure 3:**
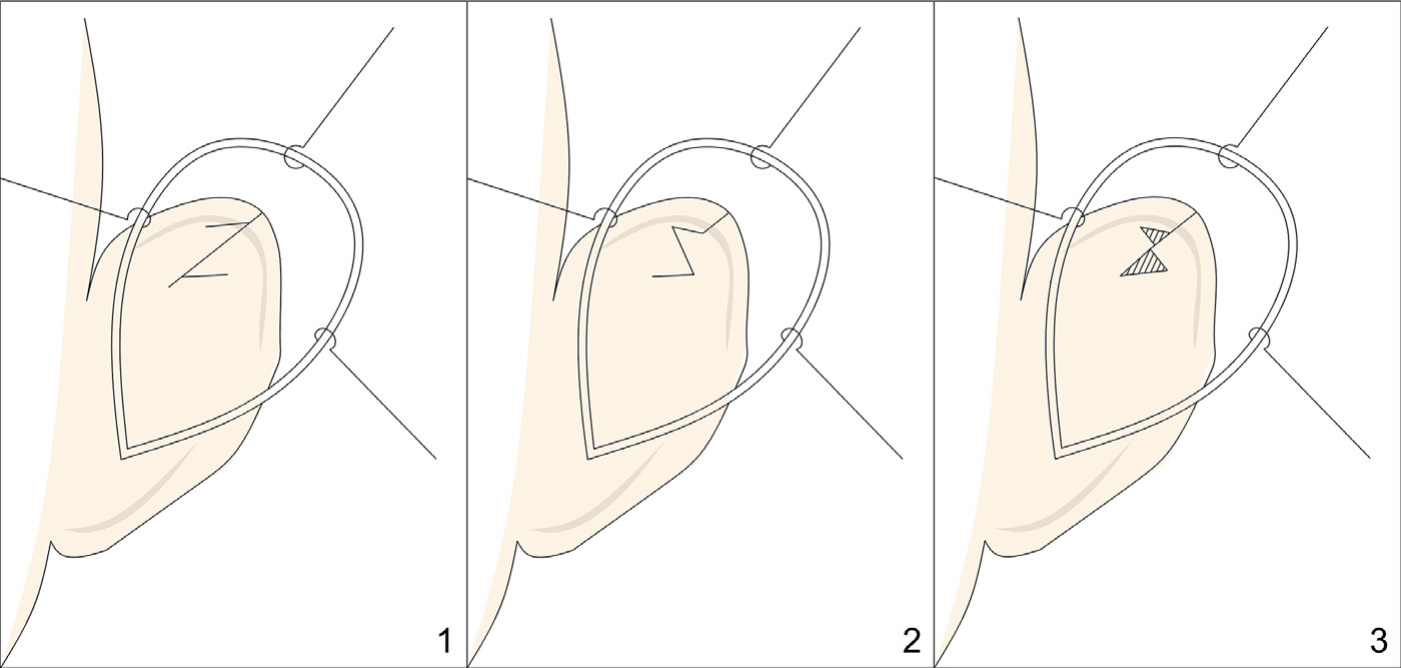
Figure depicting Nakajima’s technique. Panels 1 and 2 depict a Z-plasty of the auricular cartilage, with the central limb of the Z corresponding to the third crus. Panel 3 shows that in cases of pronounced deformity, a complete Z-plasty is not performed; instead, the tips of the triangular flaps are sutured together, creating a defect (marked with hatching) that is then covered with a perichondrial graft.

Nakajima^13^ proposed another technique where the cartilage is incised and reshaped to correct the deformities. The postauricular skin is incised and undermined to expose the anterior upper third of the helix. Depending on the severity of the deformity, three surgical options are available (Figure 3). For mild deformities, a Z-plasty incision is performed, with the third crus forming the central arm of the Z. In cases of more severe deformity, the tips of the triangular flaps are sutured together, and the resulting defect is covered with a perichondrial graft. In the most severe forms, the third crus is excised, reversed, and grafted back into its original position to flatten the scapha. Following this initial step, scoring is performed on the posterior surface at the position of the superior crus of the antihelix, and mattress sutures are placed to create the upper crus.

Nakayama’s^14^ technique for correcting Stahl’s ear involves manipulating the ear by pressing the cymba posterosuperiorly while moving the helix in the opposite direction, which helps to improve the deformity. This method is based on the finding that altering the direction of the cymba can optimize correction. Initially, the cymba is pressed posterosuperiorly to determine the ideal direction for deformity correction, and the axis of correction, along with the target position for the upper crus, is marked.

A posterior incision is made just behind the cephaloauricular groove, followed by posterior dissection. A periosteal graft is harvested from the temporal bone, and several posterior incisions are made along the position of the superior crus. Horizontal mattress sutures are then placed to create the superior crus. The cymba is subsequently sutured posterosuperiorly to the temporal fascia along the predetermined axis, a maneuver that flattens the third crus.

To address the helical rim deformity, a tunnel is created from the anterior surface of the helical rim to the posterior surface, following the base of the superior crus and traversing the cartilage via a stab incision. The periosteal graft is threaded through this tunnel and sutured to the cartilage, providing sufficient tension to bend the helical rim and correct the deformity.

**Figure 4:**
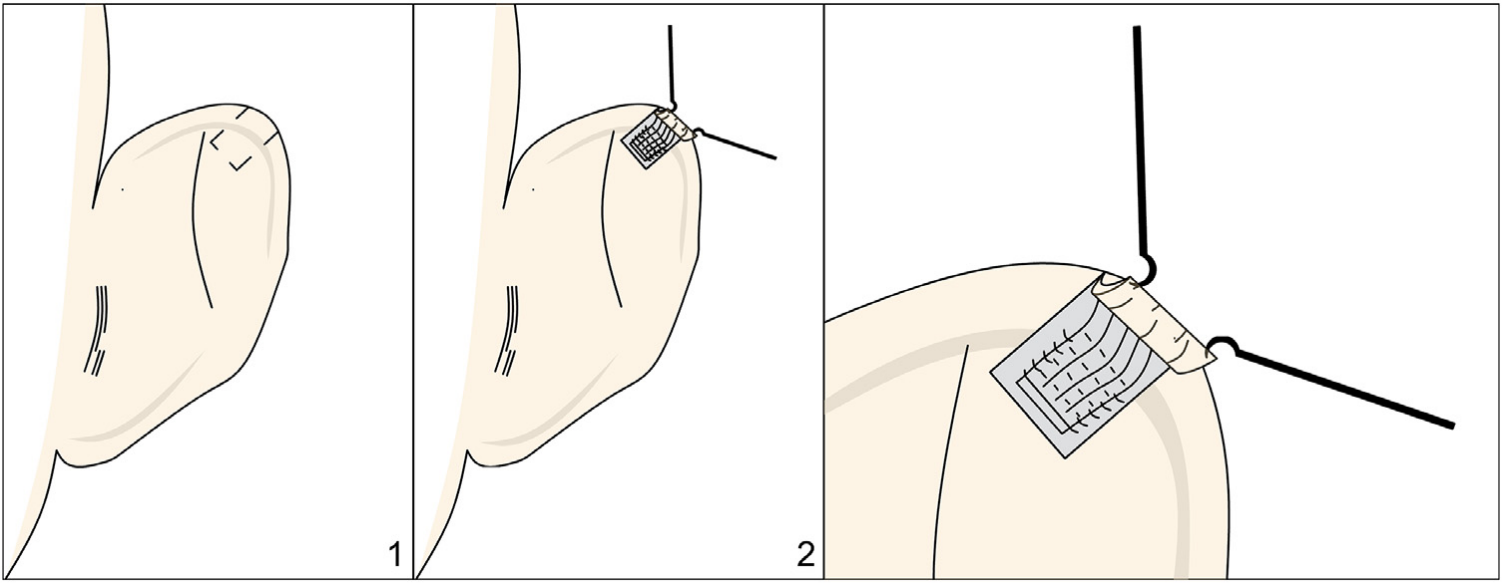
Figure depicting Tsujiguchi’s technique. Panel 1: Postauricular rectangular incision. Panel 2: Advancement of a cartilage flap toward the helix.

In a method from Tsujiguchi,^15^ a posterior skin incision is made, followed by dissection to the helical margin. A rectangular incision is created in the cartilage, incorporating the third crus but leaving the flap hinged at the helical margin (Figure 4). The cartilage is then scored, and the flap is sutured in a more advanced position toward the helical rim, leaving the most distal portion of the initial rectangular incision without cartilage. This technique does not excise the third crus; instead, it utilizes the third crus as a flap, with cartilage scoring and advancement toward the helical rim.

**Figure 5:**
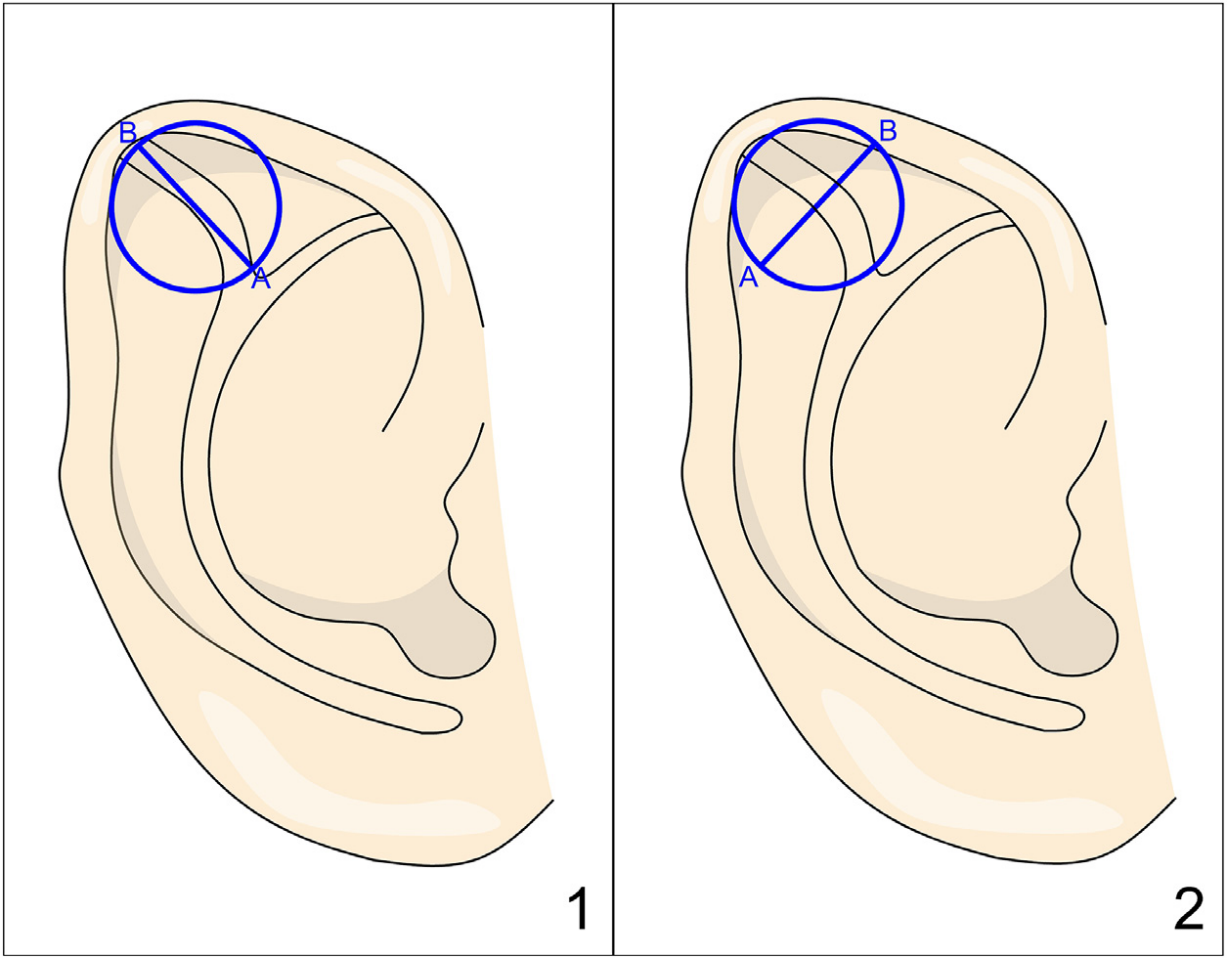
Figure depicting Sugino’s technique. Panel 1: Excised cartilage, represented by the blue disk. Panel 2: The cartilage is turned over, rotated, and then reinserted into its original position.

In Sugino’s^16^ method, two points are first marked on the third helical crus: one at the intersection with the antihelix and the other 1–2 mm from the helical rim. A circle, with a diameter matching the distance between these points (AB), is then drawn on both the anterior and postauricular surfaces using tattooing for precise marking (Figure 5). A postauricular incision and subcutaneous dissection are performed, and the marked circular section of cartilage is excised. This cartilage piece is then turned over and rotated so that line AB aligns with a line parallel to the scaphoid fossa groove, and it is sutured into place.

Min Kim^17^ described a 4 cm posterior incision extending from the helical root to the lateral end of the helical rim. A subcutaneous posterior dissection is then carried out down to the third crus. The additional crus is scored to reduce its strength. Subsequently, a piece of cartilage is harvested from the concha and placed as an onlay graft on the scapha, acting as an anatomical splint to enhance its shape.

### Cartilage-Cutting techniques

The cartilage-cutting techniques for correcting Stahl's ear involve excising portions of the cartilage to reshape the ear and correct the deformities.

**Figure 6:**
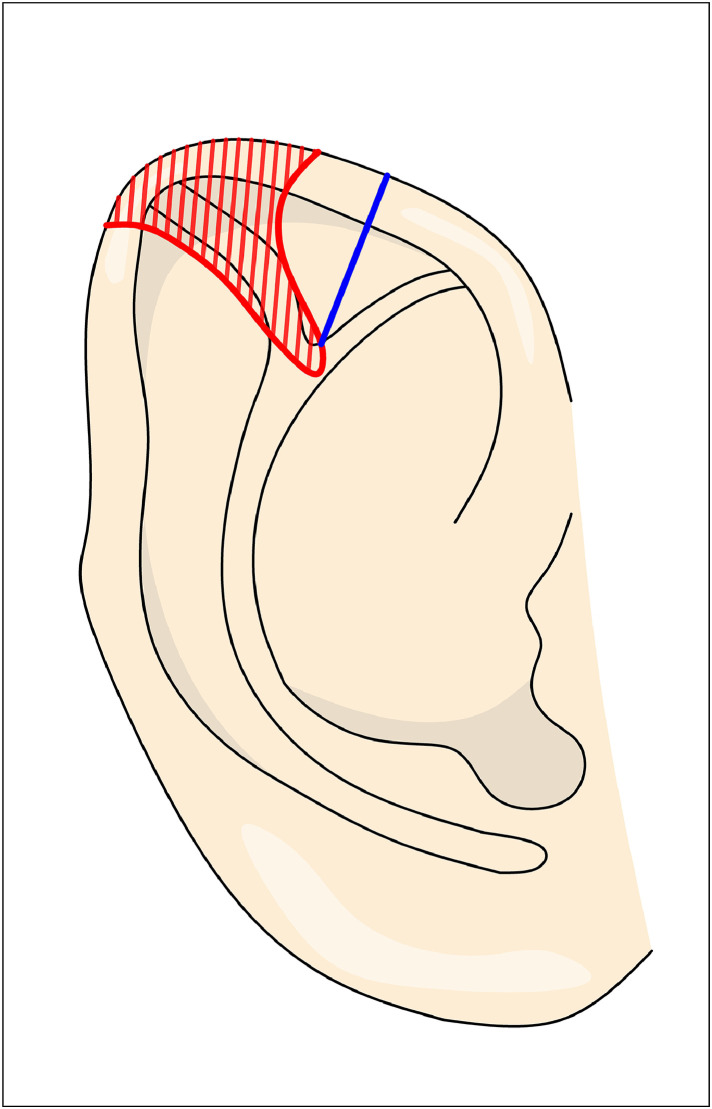
Figure depicting Yamada’s technique. Excision of the cartilage in the hatched red area, corresponding to the third crus and deformed helix. The superior crus is created along the blue line using horizontal mattress sutures.

In Yamada’s^4^ technique, a posterior incision is made parallel to the helix, followed by subperichondrial dissection. A 1 mm strip of cartilage is excised from the third crus followed by suture repair with horizontal mattress sutures to eliminate the convexity of the scapha (Figure 6). Then a wedge of the deformed helix is excised so the helical cartilage can fold anteriorly. Finally, horizontal mattress sutures are needed to shape the superior crus (with cartilage incision if needed). For type 3 Stahl's ear, Yamada specified that an elliptical excision of the cartilage, including the third crus, is performed. The excised cartilage is then turned over and sutured back into the same location for better correction.

Ono’s^18^ technique involves a similar pattern of cartilage resection, but the skin incision differs along with the addition of a cartilage graft.

The technique begins with marking the incision lines along the borders of the third crus for excision. A wedge-shaped incision is made around the abnormal third crus, removing both the deformed cartilage and the anterior skin while preserving the posterior skin. The incision extends to the flattened helix, ensuring that the wedge excision is wider in the helix area than in the scapha. In the helix, both the abnormal flattened cartilage and anterior and posterior skin are removed.

Next, relaxing incisions are made between the scapha and the normal helix, extending 5 mm on each side. Additional cartilage, harvested from the concha, is then secured to the posterior surface of the scapha cartilage and sutured in place to create a normal, concave scapha. Finally, the helix is sutured to establish a natural contour, completing the reconstruction of a normal ear shape.

**Figure 7:**
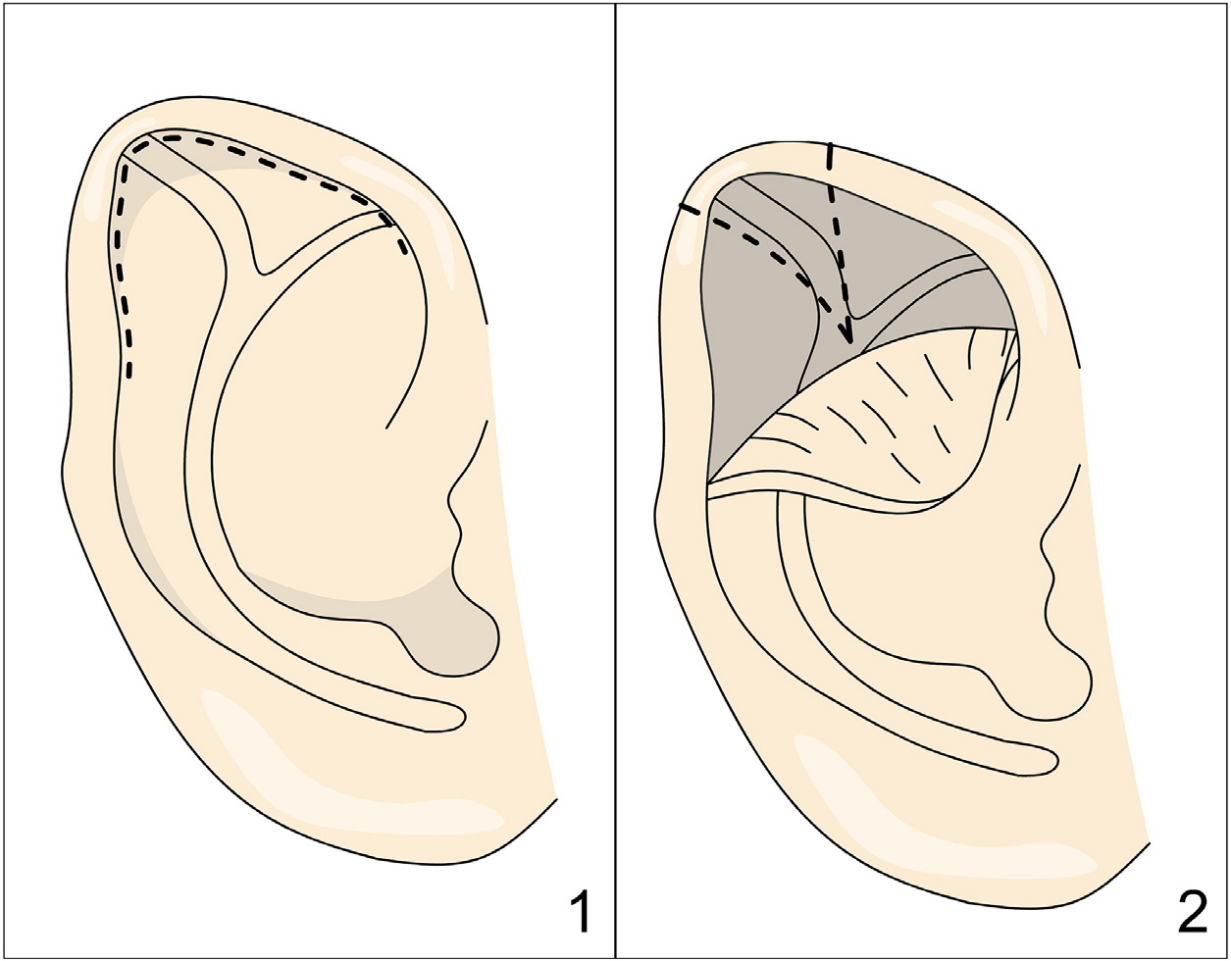
Figure depicting Kaplan’s technique. Panel 1: Incision line at the anterior helical rim. Panel 2: After elevation of the anterior skin flap, a wedge-shaped excision of the third crus and the deformed helix, along with the postauricular skin, is performed.

Kaplan^19^ described a technique in which an incision is made along the helical border on the anterior skin, followed by dissection to elevate an anterior skin flap. A wedge excision of the third crus cartilage, along with the underlying posterior skin, is then performed (Figure 7). The posterior skin and cartilage are sutured, and an onlay graft, fashioned from the excised cartilage, is placed in the correct superior crus position to reshape the structure. The graft is secured with 6–0 nylon sutures.

Borrelli’s^20^ technique shares many similarities with Kaplan’s technique. A similar anterior helical rim incision is made with dissection to expose the cartilaginous framework. A V-shaped excision of the third crus is then performed, with the cartilage approximated using posteriorly everted sutures. (Unlike Kaplan’s technique, the posterior auricular skin is not excised). In the second step, anterior horizontal mattress sutures are placed to create the superior crus. Finally, the skin is redraped without excessive trimming.

Gundeslioglu’s^21^ technique is similar in terms of skin incision and cartilage exposure. However, instead of completely excising the third crus cartilage, it is transposed as a flap to form the superior crus.

An anterior skin flap is elevated through a helical rim incision, exposing the third crus and the intended position of the superior crus. Two parallel incisions are made on either side of the third crus cartilage to create a cartilage flap, leaving it attached inferiorly. An incision is then made at the desired location of the superior crus. The cartilage flap is transposed, folded, and sutured into the area of the superior crus to reconstruct its contour. Finally, excess skin and cartilage at the helical rim are excised correcting the helical rim deformity.

**Figure 8:**
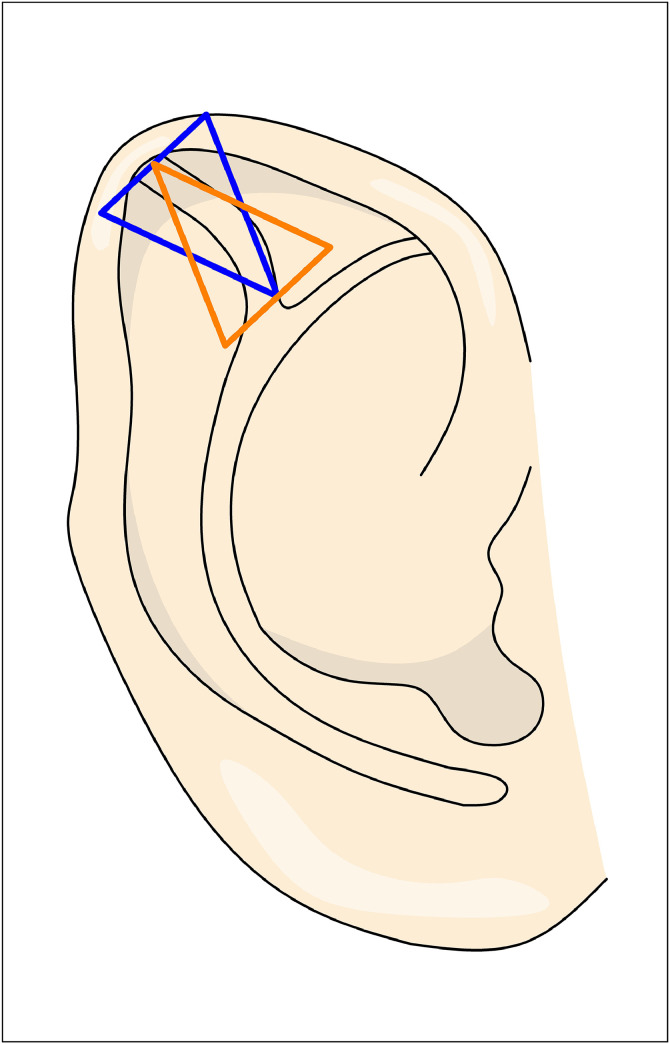
Figure depicting Sinnott’s technique. The orange triangle represents the cartilage wedge excision, while the blue triangle indicates the excision of excess anterior skin.

Sinnott’s^2^ technique involve a postauricular incision, followed by a subcutaneous dissection. The third crus cartilage is identified and excised in a wedge-shaped fashion, with the edges approximated (Figure 8). The cartilage wedge is broader medially and tapers laterally at the helical border, unlike the cartilage excision described by Ono and Yamada.

Horizontal mattress sutures are then placed to create the superior crus in the previously marked position. Additionally, Furnas sutures are used from the concha to the mastoid fascia to correct auricular prominence. Finally, any excess anterior skin resulting from the cartilage wedge excision is marked and removed as a wedge, with the apex opposite to that of the cartilage wedge. The skin is then closed.

**Figure 9:**
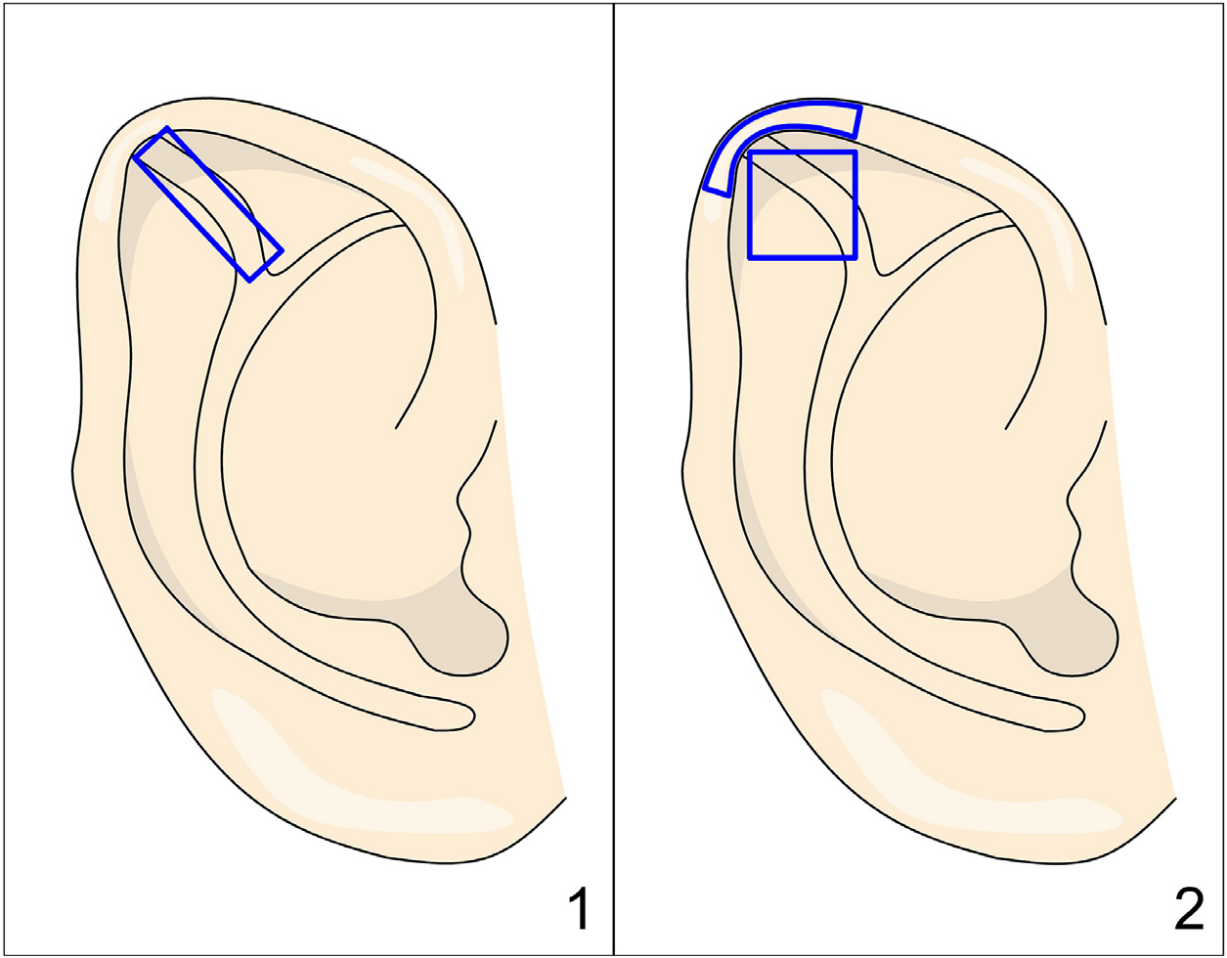
Figure depicting Al Qattan’s technique. Panel 1 shows a rectangle representing the excised cartilage, which is then grafted at the helical rim. Panel 2 displays the grafted cartilage at the helical rim, along with a square graft placed at the scapha, harvested from the concha.

Al Qattan’s^22^ technique consists of a postauricular sulcus incision, followed by subcutaneous dissection. The cartilage forming the third crus is excised and reshaped to support and contour the helical rim, to which it is then sutured. Additionally, cartilage is harvested from the concha, shaped appropriately, and used to reconstruct the scapha defect (Figure 9).

Finally, Liu’s^23^ technique is described by the author as both a cartilage-cutting and cartilage-sparing method although no cartilage excision is performed.

First, the desired position of the superior crus is marked on the anterior surface of the ear. A line 3–5 mm posterior to this position, corresponding to the cartilage cutting line, is transposed posteriorly using a needle. A posterior elliptical skin incision is then made, followed by a full-thickness cartilage incision along the premarked line. Anterior subcutaneous dissection is carried out to the medial border of the desired superior crus. Posterior cartilage scoring is performed, after which the cartilage flap is folded and sutured to the underlying cartilage, creating the superior crus in its desired position. Finally, posterior dissection is performed to expose the helical cartilage, which is rotated and shaped to form a normal helical contour.

## Discussion

This review stands as the first comprehensive analysis of the diverse surgical techniques developed for correcting Stahl's ear, offering a detailed comparison to guide clinical decision-making. Table 2 provides an overview of the advantages and limitations associated with each technique, aiding surgeons in selecting the most suitable approach for individual cases.

**Table 2:** Advantages and limitations of surgical techniques for correcting Stahl's ear deformity.

Technique	Advantage	Limitation	Cartilage cutting/sparing
Furukawa	. Helical rim corrected	. Unable to flatten third crus . Unable to create superior crus	Sparing
Weinfeld	. Correct helical contour . Enhance superior crus . Eliminate third crus	. Anterior scar on the ear . Not applicable in square shaped and wide triangular fossa	Sparing
El Kollali	. No scars anteriorly . No cartilage excision	. Helical rim irregularity may persist . Not useful in stiff third crus . Best done with no helical involvement	Sparing
Kazi	. Posterior hidden incision	. Not applicable if cartilages stiff	Sparing
Noguchi	. Simple technique splinting	. Requires prolonged ear splinting	Sparing
Nakajima	. Variety of options based on third crus severity	. Cartilages may not be suitable for Z plasty . May result in buckling and suturing under tension	Sparing
Nakayama	. Corrects all aspect of Stahl’s ear	. Requires experience to adjust the tension of the string . Requires harvesting a periosteal graft from the mastoid area	Sparing
Tsujiguchi	. Simple procedure . Correct the third crus deformity and deepen the helical sulcus	. No shaping of superior crus	Sparing
Sugino	. Simple procedure	. Might not be able to correct helical deformities . More suitable for cases where the third crus is not excessively acute	Sparing
Min Kim	. Cartilage scapha graft allows for effective use even in cases with stiff cartilage	. Need for conchal cartilage harvest . Unable to create superior crus	Sparing
Yamada	. Corrects all aspect of Stahl’s ear	/	Cutting
Ono	. Corrects helical rim deformity . Good cartilage exposure with anterior incision . Cartilage grafts serve as structural support for the scapha	. Anterior scar on the ear . Superior crus reconstruction not mentioned	Cutting
Kaplan	. Use of cartilage graft from excised cartilage . Relatively straightforward procedure . Does not rely on sutures	. Anterior scar on the ear . Reduced ear size	Cutting
Borrelli	. Good cartilaginous exposure	. Anterior scar on the ear	Cutting
Gundeslioglu	. Reduce scapha size . Helical fold creation . Corrects all aspect of Stahl’s ear	. Anterior scar on the ear	Cutting
Sinnott	. Excised cartilage wedge narrower laterally, which helps prevent excessive reduction in ear size and reduces the risk of dehiscence	. Anterior scar on the ear	Cutting
Al Qattan	. No anterior scar . Simple technique	. Need for conchal cartilage harvest . Helical rim deformity noted . No shaping of superior crus	Cutting
Liu	. Reduce scapha size if size increased . Corrects all aspect of Stahl’s ear	/	Cutting/sparing

For patients diagnosed with Stahl's ear early in life, non-surgical ear molding should be attempted, as it has a high success rate for correcting the deformity.^6^^,^^24^^,^^25^ Healthcare providers must be well-informed about the early detection and effective management of Stahl’s ear deformity. Parents should also receive guidance on the correct application of ear splints. Promptly referring diagnosed cases to a plastic surgeon for appropriate intervention is essential. However, in cases where non-surgical therapy is unsuccessful or when diagnosis occurs later in childhood, surgical intervention becomes necessary.

### Technique selection

In selecting a surgical approach for Stahl's ear correction, it is essential to tailor the technique to each individual patient, as the ideal method can vary significantly based on several key factors.

**Figure 10:**
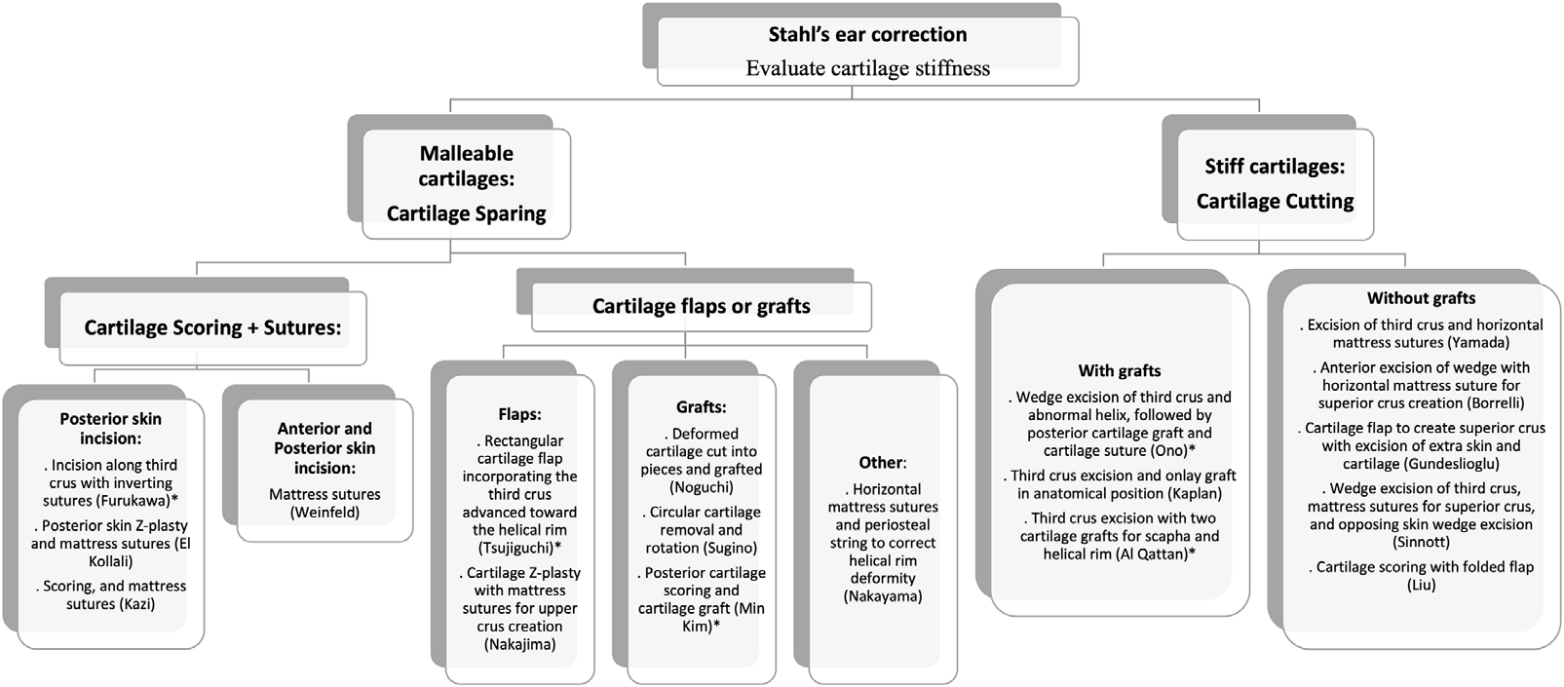
Algorithm designed to aid in clinical decision-making for Stahl’s ear correction.

To aid in clinical decision-making, we created an algorithm to guide the choice of the procedure (Figure 10). The unique morphology of the ear, including the specific characteristics of the deformity, plays a critical role in guiding the choice of technique. Additionally, the malleability of the ear cartilage affects the degree to which the cartilage can be reshaped and stabilized, influencing the surgeon’s choice of method. The size of the ear and scapha are also important considerations, as these anatomical factors may dictate the feasibility and effectiveness of certain approaches. Finally, the surgeon’s familiarity and experience with each technique can impact both the precision of the correction and the final aesthetic outcome. Therefore, a patient-centered approach, where the method is chosen based on the individual’s unique anatomy and the surgeon’s expertise, is essential for optimizing results in Stahl ear correction.

*: Indicates techniques that do not create a superior crus.

The selection of surgical technique for Stahl's ear correction should first consider the specific deformities present in each case, as some techniques address only certain aspects of the malformation. For example, Furukawa’s method may be insufficient in cases with a prominent third crus, as it does not fully flatten this structure. Similarly, the techniques developed by Ono, Tsujiguchi and Al Qattan do not reconstruct a superior crus, limiting their applicability to cases where a superior crus is already present. Additionally, some approaches, such as those by El Kollali, Sugino and Kazi do not address helical rim deformities, making them suitable only when helical rim irregularities are absent or minimal. Also, when patients present with an increased auricular scapha size, techniques reducing its size are beneficial to improve the aesthetic outcome as the techniques described by Liu, Gundeslioglu and Ono.

Tailoring the technique to the specific deformities present in each patient’s ear is therefore essential for achieving optimal outcomes.

After analyzing the specific deformities present and selecting an appropriate technique, it is also crucial to assess cartilage malleability, as this determines the suitability of either cartilage-sparing or cartilage-cutting approaches. Cartilage-sparing methods are particularly useful for avoiding contour irregularities that can sometimes arise with cartilage-cutting techniques. However, with stiffer cartilage, cartilage-sparing approaches may not achieve sufficient correction, as the cartilage's natural elasticity remains intact. In such cases, cartilage-cutting techniques become necessary to achieve the desired reshaping. Often, surgeons will combine both cutting and sparing methods to optimize correction, balancing structure with a smooth, natural contour.

Practically speaking, when patients have flexible cartilage, we prefer cartilage-sparing methods, as these approaches respect the integrity of the cartilage by avoiding removal, which contributes to stable, natural results. Techniques such as scoring and suturing the cartilage can effectively restore a normal ear appearance by eliminating the third crus, forming a superior crus, and creating a smoothly folded helical contour. Examples of cartilage-sparing techniques include those described by Furukawa, Nakajima, Nakayama, Noguchi, Tsujiguchi, and Weinfeld. Conversely, in cases where cartilage is stiff, scoring and suturing alone may not provide sufficient correction or ensure lasting results. In these instances, excision of the abnormal cartilage becomes necessary to achieve a stable and accurate contour. Techniques that incorporate cartilage excision for stiffer cartilage include those described by Yamada, Kaplan, Ono, Sinnott, Al Qattan, and Borrelli.

Many articles on Stahl’s ear correction often lack long-term outcome data, highlighting the need for further research to assess the durability and success of various treatment methods over time. Additionally, comparative studies are essential to determine which techniques are most effective in correcting the deformities and providing the best aesthetic and functional results for patients. However, given the relatively low prevalence of this condition, conducting such studies can be challenging.

## Conclusion

This study presents the first comprehensive review of surgical techniques for correcting Stahl’s ear deformity, providing a detailed analysis of their advantages, limitations, and clinical indications. By consolidating available knowledge, it offers surgeons a practical guide to evaluate and compare techniques, enabling the selection of the most appropriate approach tailored to each patient’s unique anatomy and presentation. This review aims to improve surgical decision-making and enhance patient outcomes.

## Funding

No funding was received for the research, authorship, and publication of this article.

## Ethical approval

Not required.

## Declaration of competing interest

The authors have no financial disclosures to declare. There were no potential conflicts of interest with respect to the research, authorship, and publication of this article.

## References

[1] Aki F.E., Kaimoto C.L., Katayama M.L., Kamakura L., Ferreira M.C. (2000). Correction of Stahl’s ear. Aesth Plast Surg.

[2] Sinnott C.J., Boutros C., Davenport T.A., Ruotolo R.A. (2019). The double-reverse wedge excision technique: a novel approach to reconstruction of Stahl’s Ear deformity. Plastic Reconstruct Surg Glob Open.

[3] Gleizal A., Bachelet J.T. (2013). Aetiology, pathogenesis, and specific management of Stahl’s ear: role of the transverse muscle insertion. Br J Oral Maxillofac Surg.

[4] Yamada A., Fukuda O. (1980). Evaluation of Stahl’s ear, third crus of antihelix. Ann Plast Surg.

[5] van Wijk M.P., Breugem C.C., Kon M. (2009). Non-surgical correction of congenital deformities of the auricle: a systematic review of the literature. J Plast Reconstr Aesthet Surg.

[6] Charipova K., Rogers A., Nigam M., Kotha V.S., Barra C., Baker S.B. (2020). Do not stahl: analyzing 10-year trends of nonsurgical ear molding as early intervention for congenital ear anomalies. Plastic Reconstruct Surg Glob Open.

[7] Petersson R.S., Friedman O. (2008). Current trends in otoplasty. Curr Opin Otolaryngol Head Neck Surg.

[8] Furukawa M., Mizutani Z., Hamada T. (1985). A simple operative procedure for the treatment of Stahl’s ear. Br J Plast Surg.

[9] Weinfeld A.B. (2012). Stahl’s ear correction: synergistic use of cartilage abrading, strategic Mustarde suture placement, and anterior anticonvexity suture. J Craniofac Surg.

[10] El Kollali R. (2009). Posterior Z-plasty and J-Y antihelixplasty for correction of Stahl’s ear deformity. J Plast Reconstr Aesthet Surg.

[11] Kazi A.A., Hirsch S.D., Petersson R.S. (2020). Surgical correction of Stahl ear using cartilage-cutting and -sparing techniques. Otolaryngology Case Reports.

[12] Noguchi M., Matsuo K., Imai Y., Furuta S. (1994). Simple surgical correction of Stahl’s ear. Br J Plast Surg.

[13] Nakajima T., Yoshimura Y., Kami T. (1984). Surgical and conservative repair of Stahl’s ear. Aesth Plast Surg.

[14] Nakayama Y., Soeda S. (1986). Surgical treatment of Stahl’s ear using the periosteal string. Plast Reconstr Surg.

[15] Tsujiguchi K., Tajima S., Tanaka Y., Hira M. (1992). A new method for correction of Stahl’s ear. Ann Plast Surg.

[16] Sugino H., Tsuzuki K., Bandoh Y., Tange I. (1989). Surgical correction of Stahl’s ear using the cartilage turnover and rotation method. Plast Reconstr Surg.

[17] Kim S.M., Kwon B.Y., Jun Y.J., Kim Y.J. (2017). Innovative method to correct Stahl ear that involves full thickness scoring incisions and an onlay graft of cymba conchal cartilage. Br J Oral Maxillofac Surg.

[18] Ono I., Gunji H., Tateshita T. (1996). An operation for Stahl’s ear. Br J Plast Surg.

[19] Kaplan H.M., Hudson D.A. (1999). A novel surgical method of repair for Stahl’s ear: a case report and review of current treatment modalities. Plast Reconstr Surg.

[20] Borrelli M.R., Davidson E.H., Kumar A.R. (2017). A Novel Three-Step Method for Correction of Type 1 Stahl Ear. J Craniofac Surg.

[21] Gundeslioglu A.O., Ince B. (2013). Stahl ear correction using the third crus cartilage flap. Facial Plast Surg.

[22] Al-Qattan M.M., Hashem F.K. (2004). An alternative approach for correction of Stahl’s ear. Ann Plast Surg.

[23] Liu L., Pan B., Lin L., Yu X., Yang Q., Zhao Y. (2011). A new method to correct Stahl’s ear. J Plast Reconstruct Aesthet Surg.

[24] Ullmann Y., Blazer S., Ramon Y., Blumenfeld I., Peled I.J. (2002). Early nonsurgical correction of congenital auricular deformities. Plast Reconstr Surg.

[25] Panopoulou G., Petrou I., Vassiliou A. (2022). Nonsurgical Correction of Stahl’s ear in neonates: a case study. Plastic Reconstruct Surg Glob Open.

